# Gaps in disaster cost reporting and rising economic burdens in Canada, 1990–2020: retrospective database analysis

**DOI:** 10.1016/j.lana.2026.101445

**Published:** 2026-03-20

**Authors:** Mazen El-Baba, Attila J. Hertelendy, Amalia Voskanyan, Gregory R. Ciottone, Fadi Issa

**Affiliations:** a BIDMC Disaster Medicine Fellowship, Department of Emergency Medicine, Beth Israel Deaconess Medical Center and Harvard Medical School, Boston, MA, USA; b Division of Emergency Medicine, Department of Medicine, University of Toronto, Toronto, Ontario, Canada; c Department of Information Systems and Business Analytics, College of Business, Florida International University, Miami, FL, USA

**Keywords:** Disaster preparedness, Health equity, Wildfires, Climate change, Health systems resilience

## Abstract

**Background:**

Disasters increasingly threaten population health and health-system resilience. Yet, the economic costs that inform preparedness and recovery remain unevenly measured. Missing cost data, particularly for rural and Indigenous communities, may contribute to inequities in disaster funding and response. We aimed to characterize disaster-related cost reporting and temporal trends in Canada (1990–2020), and to examine patterns of missing data across community contexts.

**Methods:**

We conducted a retrospective database analysis of federally recognized disasters in Canada (1990–2020) using the *Canadian Disaster Database*, analyzing reported direct economic losses from a national, mixed-public-sector perspective. Reported costs were adjusted to 2020 Canadian dollars using Consumer Price Index values. Events were grouped by hazard, and temporal trends in log-transformed, inflation-adjusted costs were assessed using ordinary least squares regression. Scenario-based bounding analyses illustrate the possible range of missing costs. Events were further classified as *urban/metro* or *rural and/or Indigenous* to evaluate differences in cost reporting.

**Findings:**

Between 1990 and 2020, meteorological (n = 402) and wildfire (n = 90) disasters accounted for most recorded losses in Canada, totalling CAD $38.3 billion (2020 value). Inflation-adjusted costs increased by 4.5% per year for meteorological events and 25.6% per year for wildfires. Among 636 total disaster events, only 278 (44%) had reported costs, revealing data gaps, with reporting completeness varying substantially over time. Cost reporting was lower for disasters affecting rural and Indigenous communities compared with urban/metro areas (56.0% vs 31.6%).

**Interpretation:**

Disaster costs in Canada are rising, driven by meteorological and wildfire events, yet more than half of recorded disasters lack cost data. These data gaps reflect limitations in disaster cost surveillance and reporting systems, with lower reporting observed for disasters affecting rural and Indigenous communities. Strengthening standardized and equity-sensitive disaster cost reporting should be recognized as a public-health and health-systems priority in an era of climate change.

**Funding:**

None.

**Research in context**

**Evidence before this study**

During initial scoping of national data sources, we examined the Canadian Disaster Database (CDD), the primary federal repository of disaster events, and observed substantial missingness in reported cost fields. This prompted a targeted search on disaster cost measurement and reporting in Canada. We searched PubMed and Google Scholar to identify evidence on the economic impacts of disasters, focusing on whether costs have increased over time for hazards most consequential in Canada, including meteorological events and wildfires. We also searched for literature relevant to geographic and community-context differences in disaster impacts, including rural and Indigenous communities. We used the Google search engine to identify grey literature (government and agency reports and policy documents) relevant to disaster management, disaster risk reduction, and disaster loss accounting in Canada and internationally. The initial search was conducted from July 10, 2025, to Sept 20, 2025, and an updated search to inform manuscript revision was conducted from Dec 6, 2025, to Dec 15, 2025. Search terms combined Canada OR Canadian with disaster OR wildfire OR flood∗ OR storm∗ OR meteorolog∗ OR “climate change∗”, and cost OR “economic loss∗” OR damage∗, with additional terms rural OR remote OR Indigenous OR “First Nations” OR Inuit OR Métis. Overall, we found hazard-specific Canadian and international studies describing rising disaster impacts, but no national, event-level analysis that examined cost trends in Canada alongside cost reporting completeness over time and across community contexts.

**Added value of this study**

We provide a national, hazard-level (meteorological vs wildfire) assessment of disaster cost reporting in Canada using the Canadian Disaster Database (1990–2020). We describe the distribution of reported direct losses across hazards, assess inflation-adjusted cost trends for meteorological disasters and wildfires, and quantify cost-reporting completeness overall and over time. Using scenario-based bounds, we show how missing cost data can materially alter cumulative national loss estimates. We also compare reporting completeness across community contexts (urban/metropolitan vs rural and/or Indigenous), highlighting how uneven cost documentation can have equity implications for disaster preparedness and recovery.

**Implications of all the available evidence**

In Canada, climate-related hazards and their impacts are increasing, yet disaster loss accounting remains incomplete and difficult to compare across time and jurisdictions. We show that more than half of Canadian disasters recorded in a federal repository lack cost estimates, which can distort trend interpretation and hazard ranking. We find lower reporting completeness for events affecting rural and/or Indigenous communities. Such under-documentation may reinforce inequities and risks reducing the visibility of impacts in settings where vulnerability and recovery constraints are often greatest, with downstream consequences for preparedness prioritisation, recovery planning, and accountability. Strengthening national disaster cost surveillance will require clearer standards for cost estimation and reporting, greater transparency about how estimates are derived, and equity-attentive documentation practices so that all communities are consistently visible in national data systems.

## Introduction

The health and economic consequences of disasters are rising globally, driven by increasing hazard severity, population exposure, and longstanding gaps in preparedness.^1^^,^^2^ Across the Americas, wildfires, floods, and severe storms have grown more frequent and destructive; exerting substantial pressure on emergency management and health systems.^3^^,^^4^ Canada's vast geography and climatic diversity make it particularly vulnerable to meteorological (floods, droughts, severe temperatures, tornados, and storms) and wildfire disasters.^5^^,^^6^ Despite increased global losses due to these disasters,^7^ no comprehensive assessment has systematically examined reported disaster-related economic losses and the completeness of cost reporting in Canada. Such analyses are essential to guide preparedness policy, inform equitable allocation of resources, and evaluate whether current investments are sufficient to reduce future risks.

Globally, economic losses from meteorological and wildfire disasters have escalated due to both climate change and expanding development in high-risk regions.^8^^,^^9^ Reported disaster costs typically capture direct damages to infrastructure and communities, yet they omit many indirect and longer-term impacts, including displacement,^10^ disruption of healthcare systems,^11^, ^12^, ^13^ diversion of scarce resources,^14^ and downstream economic consequences.^15^^,^^16^ Although total disaster losses (direct and indirect) can be estimated using economic modeling approaches, such methods require detailed, component-level loss data and consistent reporting over time.^17^^,^^18^ Nonetheless, direct cost estimates remain a valuable proxy for disaster magnitude and allow for systematic tracking of trends.

In Canada, the need to understand disaster costs is amplified by longstanding healthcare system constraints. Hospitals and emergency departments have operated at or beyond capacity for decades,^19^^,^^20^ leaving little reserve to absorb disaster-related surges.^21^^,^^22^ These strains magnify the indirect costs of disasters, as resources are diverted and service disruptions extend beyond the immediate event. Importantly, these burdens are not evenly distributed: rural and Indigenous communities, which already face chronic healthcare access barriers, greater geographic exposure,^22^ limited evacuation routes,^23^^,^^24^ and lower insurance and infrastructure coverage^25^ are disproportionately affected by wildfires and floods, and face additional challenges in evacuation, recovery, and continuity of healthcare.^26^, ^27^, ^28^, ^29^ These inequities heighten vulnerability to meteorological and wildfire events and reflect broader structural determinants that influence disaster preparedness and recovery.

Responsibility for health and emergency management in Canada is shared across jurisdictions: provincial and territorial governments lead operations, while federal agencies provide funding and surge support.^30^ Indigenous leadership also plays a central role, particularly in self-governing communities and regions frequently affected by disasters. Recent policy debates around wildfire management have underscored the limitations of this decentralized model and the need for stronger national coordination.^31^ Establishing robust cost analysis is critical to support equitable preparedness planning^32^ and to inform discussions on whether national approaches should extend beyond wildfire management.

We conducted a retrospective database analysis of the Canadian Disaster Database (1990–2020) to characterize disaster-related cost reporting and temporal trends, and to examine patterns of missingness across community contexts. Analyses focused on meteorological and wildfire events, which are the most consequential hazards for Canadian communities: meteorological disasters account for the majority of reported losses,^33^ while wildfires have intensified in frequency and severity in recent decades,^26^^,^^29^ with national analyses showing significant increases in high-severity burn days (0.5 additional days between 1981 and 2020), rising burn severity, and record breaking burned area, including nearly 15 million hectares in 2023.^34^ These trends have disproportionately affected rural and Indigenous populations.^35^ We aimed to characterize disaster-related cost reporting and temporal trends in Canada (1990–2020), and to examine patterns of missing data across community contexts.

## Methods

### Data source and extraction

We conducted a retrospective descriptive analysis of disasters occurring in Canada between 1990 and 2020, using data from the Canadian Disaster Database (CDD), maintained by Public Safety Canada. The CDD catalogues disasters managed by Canadian federal, provincial, or territorial authorities, documenting event type, location, date, fatalities, injuries or infections, evacuations, and estimated economic cost (in Canadian dollars). The CDD includes events that meet the Emergency Management Framework for Canada definition of a disaster, as well as events satisfying one or more of the following criteria: ≥10 deaths; ≥100 people affected, injured, evacuated, or left homeless; an appeal for national or international assistance; historical significance; or significant disruption such that local recovery is not possible without external support.

Data were accessed in August 2025 via the CDD online search tool (https://www.publicsafety.gc.ca/cnt/rsrcs/cndn-dsstr-dtbs/index-en.aspx). Events were extracted manually, as direct downloads were not available, and compiled into a structured dataset. The analysis was restricted to the period 1990–2020 as no public CDD updates have been released since 2020 and earlier records were incomplete and less temporally relevant. Events outside Canadian borders, (e.g., Canada's response to the 2010 Haiti earthquake) were excluded (9 total). Three incidents labelled “infrastructure failures” (industry/manufacturing, communication, or transportation) and two incidents labelled “civil disobedience” were removed from the analysis as they lacked sufficient information to categorize them meaningfully. Descriptive statistics for meteorological and wildfire events, including annual event counts and summary statistics for costed events (mean, median, standard deviation, and interquartile range) were computed. All analyses were conducted in Python, using standard data-analysis libraries (pandas for data cleaning, aggregation; statsmodels for regression modelling; and summary statistics and matplotlib for figure generation).

### Hazard categorization

All events were categorized based on descriptors used by the data base and were grouped into five primary hazard categories including (1) meteorological (i.e., floods, storms, tornadoes, lightning, heatwaves, and cold events); (2) Wildfire; (3) geological (i.e., earthquakes, landslides, and avalanches); (4) Biological; and (5) Chemical, Radiological, Nuclear, and Explosive events (CRNE). The “Wildfire” category was deliberately separated to allow for specific analyses given Canada's vast forested landscape and worsening wildfires in recent years.^36^ Further, wildfire's resource requirements and impacts on Indigenous and vulnerable communities differ substantially from other hazards.^26^ “Biological” events were analyzed independently from CRNE events given their differing response requirements.^37^^,^^38^

### Cost data and inflation adjustment

The study analysed reported direct economic losses from a national perspective using the CDD. According to documentation provided by Public Safety Canada, each reported “estimated economic cost” represents a composite of municipal, provincial, and federal expenditures; Disaster Financial Assistance Arrangements (DFAA) payments; insurance payouts; nongovernmental organization contributions; and other government department costs. The CDD does not publicly report disaggregated cost components. Further, reporting practices vary across jurisdictions, and no national guideline governs cost estimation or data collection. Costs were adjusted to 2020 Canadian dollars using the annual average consumer price index (CPI) from Statistics Canada (Table 18-10-0005-01, formerly CANSIM 326-0021) supplemented by the Bank of Canada historical CPI series (V41690973). For each event, the reported cost was multiplied by the ratio of the 2020 CPI to the CPI of the event year, producing standardized comparable estimates. Costs were subsequently grouped into bands for descriptive purposes to facilitate visualization of the distribution of event costs: <$1 million, $1–10 million, $10–100 million, ≥$100 million, and Not reported. These bands were used only for descriptive figures and tables and were not used in any inferential analyses.

### Scenario-based bounding analysis for missing costs

Because many events lacked reported costs and the CDD does not provide any event-level loss components or appraisal metadata, formal imputation is not feasible; instead, we applied a scenario-based bounding approach to illustrate the potential range of cumulative national costs. We used three simple scenario-based bounds (best-case, median-value, and upper-bound) to illustrate the potential range of under-reported losses. These scenarios are not intended as estimates of true costs, but rather as illustrative ranges to contextualise the magnitude of missing data. In the best-case scenario, each missing event was assigned $0.5 million (the midpoint of the <$1 million band). In the worst-case scenario, each missing event was assigned $100 million (the lower threshold of the ≥$100 million band). As a middle-ground scenario, we used hazard-specific median imputation: for each hazard category (meteorological, wildfire, geological, biological, CRNE), missing costs were imputed as the median reported cost within that category for 1990–2020. Because CRNE only had one reported cost, we used the overall median of reported events as fallback. We repeated the median-value scenario using hazard-specific medians stratified by decade and five-year bins, with fallback to the hazard-level median (1990–2020) when hazard-time strata were sparse (fewer than two costed events).

### Trend analysis

To assess temporal trends in disaster-related costs, a focused trend analysis was completed on meteorological and wildfire events between 1990 and 2020. Those two hazard categories were specifically looked at because they have the most complete data and are especially relevant to the Canadian context of disaster management and preparedness. Costs were inflation-adjusted to 2020 CAD and log transformed to reduce skewness. Trends were analyzed using ordinary least squares (OLS) regression of log 10(cost) on year, stratified by hazard category (meteorological, wildfire). The model; log10 (Cost) = α + β × Year + ϵ; where α is the intercept, β represents the annual change in log10 (cost), and ϵ is the error term. Coefficients were exponentiated (10ˆβ) to represent the multiplicative annual change in cost. To visualize potential non-linear temporal patterns, we computed centered 5-year moving averages of log10 annual costs. We additionally fit restricted cubic spline curves (three degrees of freedom) to log annual costs to assess broader trend shape.

### Per capita normalization

To account for population growth, disaster costs were standardized per capita. Costs for meteorological and wildfire events were aggregated by decade (1990s, 2000s, 2010s), divided by the number of years per decade, and expressed as annualized costs per 100,000 population. Decade-average Canadian population estimates were calculated from Statistics Canada (Table: 17-10-0005-01, formerly CANSIM 051-0001) data by averaging the annual July 1st population counts within each decade, yielding 29.12 million for the 1990s, 32.12 million for the 2000s, and 35.88 million for the 2010s (2010–2020 inclusive).

### Equity gaps in cost reporting

Meteorological and wildfire events from 1990 to 2020 were classified as affecting *urban/metro* or *rural and/or Indigenous* communities based on event location and population characteristics described in the Canadian Disaster Database. Each event was individually reviewed in detail and accompanying narrative descriptions were analyzed to determine whether additional geographic or contextual information supported classification. Events occurring in large metropolitan areas or locations designated as cities were coded *urban/metro*, whereas those in towns, rural regions, or explicitly involving Indigenous communities were coded *rural and/or Indigenous*. Events lacking sufficient geographic specificity (e.g., “Eastern Canada,” “Atlantic provinces”) were coded *unclassified* and excluded from the comparison to minimize misclassification bias. Classifications were performed without reference to cost-reporting status. Sensitivity analyses reclassified all unclassified events as *urban* and then as *rural*. Because the CDD does not provide geocodes, census linkages, or standardized socio-demographic metadata, this classification represents a coarse proxy for underlying community characteristics.

### AI-assisted coding for data processing and analysis

Large Language Model (ChatGPT, OpenAI) was used for programming assistance, such as drafting example Python code structures for data cleaning, statistical modeling workflows, and figure generation based on author-specific analytic plans and variable definitions. Code was reviewed by the authors, modified as needed, and executed locally against locally stored data. All analytical decisions, inference, and interpretation were performed by the authors.

### Ethical approval

Ethics approval was not required because this study used publicly available, non-identifiable data and involved no human participants.

### Role of the funding source

There was no funding source for this study. No funder had any role in the study design, data collection and analysis, preparation of this manuscript, interpretation of results, or the decision to submit the manuscript for publication.

## Results

### Cost reporting by hazard

**Fig. 1:**
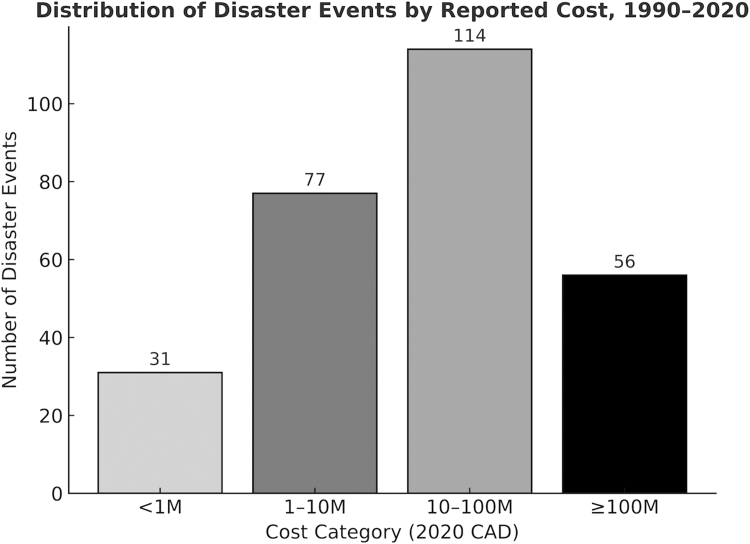
Number of disaster events with reported costs, grouped into four cost bands in 2020 Canadian dollars (CAD): less than $1 million, $1–10 million, $10–100 million, and $100 million or more. Across 278 events with reported costs, most were in the $10–100 million range, followed by $1–10 million, ≥$100 million, and <$1 million.

Between 1990 and 2020, meteorologic and wildfire events occurred a mean of thirteen and four times per year, respectively (Supplementary Table S1). Meteorologic events occurred throughout the year with higher frequencies in the spring and summer months, whereas wildfire events showed a strong seasonal concentration in summer (Supplementary Table S2). Annual counts for both hazards showed modest year-to-year variability (Supplementary Figure S1). Of the 636 disasters recorded in the Canadian Disaster Database between 1990 and 2020, 278 (44%) had reported costs and 358 (56%) lacked cost data. Cost reporting varied considerably by hazard (Table 1). Meteorological events were most frequently assigned costs, with 243 of 402 events (60%) reporting, while only 25 of 90 wildfire events (28%) and six of 16 geological events (38%) had cost estimates. Reporting was particularly limited for biological events (20%) and CRNE events (1%). Among events with reported costs (n = 278), 31 (11%) were valued at less than $1 million (2020 CAD), 77 (28%) at $1–10 million, 114 (41%) at $10–100 million, and 56 (20%) at $100 million or more (Fig. 1), underscoring the skewed distribution with a long tail of very costly disasters.

**Table 1:** Reported and missing disaster costs by hazard category, Canada, 1990–2020.

Hazard category	Total events	Events with reported costs, No. (%)	Median cost, $ millions (2020 CAD)	Mean cost, $ millions (2020 CAD)	Range, $ millions (2020 CAD)	Events with missing costs, No. (%)
Biological	15	3 (20.0%)	17.8	19.5	1.462–39	12 (80.0%)
CRNE	113	1 (0.9%)	64.7	64.7	64.707	112 (99.1%)
Geological	16	6 (37.5%)	3.1	8.0	0.275–32	10 (62.5%)
Meteorological	402	243 (60.4%)	20.1	130.2	0.056–6956	159 (39.6%)
Wildfire	90	25 (27.8%)	13.9	260.2	0.004–4341	65 (72.2%)
**Total**	636	278 (43.7%)	17.8	137.9	0.004–6956	358 (56.3%)

### Scenario-based bounding analysis for missing costs

To illustrate the potential scale of under-reporting, we applied three scenario-based bounding approaches. Observed reported costs totalled $38.3 billion. Assuming all missing events cost less than $1 million yielded an estimated cumulative total of $38.5 billion, essentially unchanged from the observed. By contrast, assigning missing events the hazard-specific median cost increased the estimate to $49.9 billion, while assuming all missing events cost $100 million or more nearly doubled the estimate to $74.1 billion. These scenarios produce cumulative estimates ranging from $38.5 billion to $74.1 billion, a difference of up to $36 billion. Time-stratified hazard-specific median scenarios (by decade and five-year bins) yielded cumulative totals of $48.0 billion and $49.8 billion, respectively.

### Focus on meteorological and wildfire events

Given their national significance and the relative completeness of cost reporting, subsequent analyses focused on meteorological and wildfire disasters. Meteorological events were the most common hazard (n = 402; 243 with reported costs), with a median cost of $20.1 million and a mean of $130.2 million, reflecting both frequent moderate-impact events and occasional high-cost floods and storms. Costs were missing for 40% of meteorological events. Wildfire events were less frequent (n = 90; 25 with reported costs) but disproportionately costly, with a median cost of $13.9 million and a mean of $260.2 million, reflecting a skewed distribution in which a small number of extreme wildfires contributed substantially to total losses. Costs were missing for 72% of wildfire events. To contextualize temporal fluctuations in reported costs, we examined the completeness of cost reporting over time (Supplementary Figure S2). The analysis shows a marked decrease in the completeness of meteorological cost reporting in the mid-2000s.

### Wildfire-meteorologic trend analysis

**Fig. 2:**
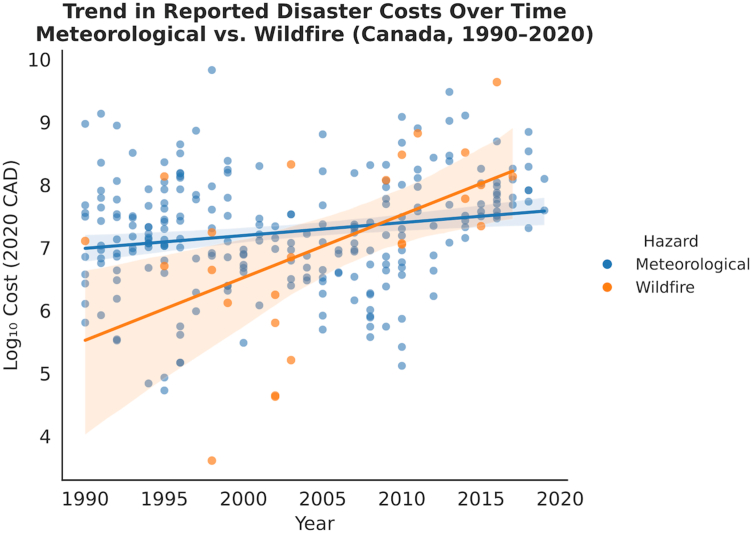
Scatterplots and ordinary least squares regression lines (95% CIs shaded) for inflation-adjusted costs (log_10_ 2020 CAD) among meteorological (blue) and wildfire (orange) events. Meteorological costs show a modest upward trend, while wildfire costs rise more steeply over time.

Temporal trends in meteorological and wildfire costs are shown in Fig. 2. Regression analysis of log-transformed, inflation adjusted costs indicated a statistically significant annual increase in costs for meteorological events (β = 0.019; 95% CI, 0.006–0.032; p < 0.01), corresponding to an approximate 4.5% increase per year. Wildfire events demonstrated a larger increase (β = 0.099; 95% CI, 0.029–0.169; p < 0.01), equating to approximately 25.6% annually. These patterns highlight a steady rise in meteorological costs and a sharper escalation in wildfire costs over time. Non-linear smoothing using both five-year moving averages and spline curves showed gradual temporal changes that were directionally consistent with the linear regression results. Wildfire costs rose steadily between 1990 and 2020, while meteorological costs displayed a mild curvature (a mid-period dip followed by an increase) without evidence of a sustained deviation from the overall upward trend (Supplementary Figure S3A and B).

### Per capita normalization

To account for population growth, disaster costs were standardized per capita. On a population-adjusted basis, annual meteorological disaster costs were approximately $5.53 million per 100,000 population in the 1990s, $0.78 million per 100,000 in the 2000s, and $3.30 million per 100,000 in the 2010s. However, reporting completeness declined substantially after the 1990s, with only about half of meteorological events in the 2000s (54%) and 2010s (47%) having reported costs, compared with more than 80% in the 1990s. The apparent dip in the 2000s coincided with lower completeness of cost reporting. In contrast, wildfire costs increased steadily, from approximately $60,000 per 100,000 in the 1990s, to $100,000 in the 2000s, and to $1.51 million per 100,000 in the 2010s. Although absolute meteorological costs remained higher overall, the per capita burden of wildfires rose markedly in the most recent decade, underscoring increasing population exposure to wildfire-related losses.

### Equity reporting analysis

**Fig. 3:**
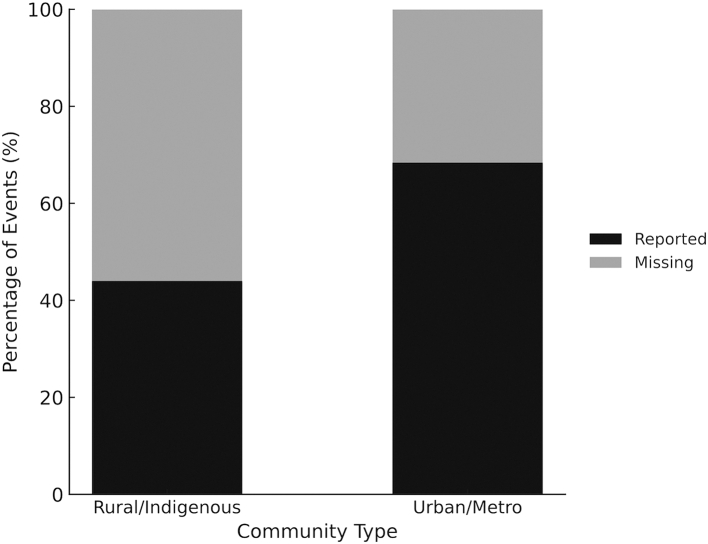
Stacked bar chart showing the proportion of disasters with reported vs missing cost data among events affecting rural/Indigenous and urban/metro communities. Costs were reported for 68.4% of urban/metro and 44.0% of rural/Indigenous events. Disasters impacting rural and Indigenous populations were nearly twice as likely to lack reported cost estimates.

Cost reporting was substantially lower for disasters affecting rural and Indigenous communities (122 of 277; 44.0%) compared with urban/metro events (67 of 98; 68.4%) (Fig. 3). Disasters impacting rural and Indigenous populations were therefore nearly twice as likely to lack reported cost estimates. Of the 117 unclassified events, 79 (67.5%) had reported costs and 38 (32.5%) were missing. Their exclusion did not alter the direction or magnitude of the observed inequities, indicating that the results were consistent across classification assumptions.

## Discussion

From 1990 to 2020, disasters in Canada resulted in tens of billions of dollars in reported economic losses, with meteorological and wildfire events accounting for most of these costs. Losses were highly skewed: most disasters caused modest damage, while a small number of extreme events accounted for the bulk of total costs. Meteorological hazards were the most frequent and consistently costly, whereas wildfires, though less common, were disproportionately expensive and showed the steepest growth over time. The overall upward trend in disaster costs is consistent with international and Canadian analyses demonstrating increasing losses driven by climate change and expanding development in high-risk areas.^9^^,^^39^^,^^40^ On a per capita basis, disaster costs rose markedly in the 2010s, consistent with climate change projections of more frequent and severe extreme weather.^41^ The apparent dip in the mid-2000s reflected missing cost data rather than true decline in impact. Further, gaps in cost data represent a systemic limitation of national disaster surveillance, shaping how temporal trends and equity patterns must be interpreted. These findings underscore the need for standardized national guidelines for disaster-loss reporting.

Meteorological and wildfire disasters often cross provincial and territorial jurisdictions, emphasizing the need for national coordination. Our findings also show that the economic burden of these disasters extends well beyond wildfires alone. Discussions around the nationalization of wildfire response represent a positive step in Canada's preparedness landscape; similar consideration is needed for other hazards. This reinforces the case for coordinated federal-provincial-territorial mechanisms, while maintaining the central role of local and regional teams in operational delivery. Against this backdrop, shifts in global risk landscapes and rising defence expenditures highlight the need to reconsider how preparedness is prioritized. Given that Canada currently allocates close to 2% of its GDP to defence, with projections approaching 5% by 2035, disaster preparedness is an essential component of national security planning.

The escalation of meteorological and wildfire disasters has direct implications for health systems.^42^^,^^43^ Beyond immediate damages, these events generate surges in emergency demand^44^^,^^45^ and contribute to long-term health consequences,^46^ compounding their total impact. Rural and northern communities, including Indigenous populations, are vulnerable due to limited surge capacity, geographic isolation, and longstanding inequities in resource allocation.^40^^,^^47^ These inequities in infrastructure, healthcare access, and disaster preparedness reflect ongoing colonial legacies that continue to influence differential health outcomes across Indigenous communities.^25^^,^^48^ Investments in disaster risk reduction and preparedness, such as floodplain management, wildfire mitigation in the wildland–urban interface, and strengthening health-system surge capacity, can lessen the health and economic impacts of extreme events.^2^ Such improvements rely on accurate longitudinal documentation and are relevant across all jurisdictions, especially rural and Indigenous communities where vulnerability is structurally higher.

The disparity in cost reporting between disasters impacting urban/metropolitan communities and those affecting rural or Indigenous communities may reflect a form of systematic bias, but should not be interpreted as evidence that this is the only form of bias within national reporting systems. This pattern is consistent with structural inequities documented in Canada, including differences in infrastructure, resource availability, and insurance penetration.^25^ In the absence of standardized national guidelines for disaster-loss reporting, multiple systemic factors may contribute to uneven documentation of economic impacts. Persistent missing-cost patterns over time (Supplementary Figure S2) suggest that under-reporting may reflect features of federal and provincial documentation processes shaped by administrative variability, inconsistent appraisal practices, differential documentation capacity, and varying levels of institutional or political prioritization. These systemic explanations operate alongside community-level inequities and together likely contribute to the observed reporting gaps. Regardless of the underlying mechanism, lower reporting among rural and Indigenous communities risks rendering the impacts of these events invisible in national data systems, reinforcing inequities in both recovery funding and health system preparedness. Improving disaster cost surveillance through equity-sensitive reporting standards is essential to ensure that the communities most affected, particularly Indigenous and rural, are visible, valued, and supported through equitable preparedness planning.

The CDD captures primarily direct economic losses and does not make underlying components of cost estimates publicly available, nor does it systematically include indirect costs. Disasters impose indirect costs, including disruptions to health services and housing,^7^^,^^49^ business interruption, environmental degradation, and long-term health and social impacts.^17^ As climate change intensifies the frequency and severity of extreme events, accounting for these indirect and longer-term impacts will be essential for accurate national risk assessment, preparedness planning, and equity-focused resilience across the Americas.^50^ Future work in Canada should focus on policy improvements to CDD reporting practices, such as making cost estimate subcomponents available, and incorporating indirect and macroeconomic impacts into disaster-loss analyses. Given that the CDD publicly reports only a single aggregated cost estimate per event, the full economic consequences of Canadian disasters are likely substantially underestimated.

Beyond these conceptual limitations, this study also has methodological constraints. Cost reporting in the Canadian Disaster Database was incomplete; as a result, our estimates almost certainly underestimate the true burden. In addition, the database is limited to events through 2020, excluding more recent disasters. This is a significant limitation given the escalating costs of recent meteorological and wildfire events, which have imposed unprecedented pressures on the economy and health systems. Further, no public documentation to our knowledge is available on data collection practices or policies that govern the CDD; therefore, potential changes in institutional capacity, data collection procedures, or cost-estimation approaches over time cannot be assessed. These constraints limit the ability to attribute missing costs to any single factor and underscore the need for greater transparency in national disaster loss accounting systems. Our examination of equity gaps is also constrained by the lack of geocoded event data, standardized socio-demographic linkages, or detailed exposure information in the CDD. The locational classification used here is thus a coarse proxy rather than a granular spatial or socio-economic measure, and some degree of misclassification is possible. Any such misclassification would likely bias estimates toward the null, meaning that the disparities we report are conservative.

Canada's experience illustrates how rising disaster costs intersect with health, equity, and national security. Embedding preparedness and equity-sensitive surveillance within health-system policy is essential to ensure that the communities most affected, particularly rural and Indigenous, are visible, supported, and resilient in the face of accelerating climate-driven risks. Preparedness as a health and equity imperative is critical to reducing the human and economic toll of disasters.

## Contributors

MEB: conceptualization, reviewed the literature, methodology, formal analysis, visualization, writing–original draft, and writing–review & editing.

AJH, FI, AV, GC: conceptualization, reviewed the literature, and writing–review & editing.

MEB and AJH directly accessed and verified the underlying data reported in the manuscript and are responsible for the decision to submit the manuscript.

All authors approved the final version.

## Data sharing statement

The Canadian Disaster Database is publicly available through Public Safety Canada. The extracted dataset used for this analysis and the analysis code are available from the corresponding author upon request.

## Use of Artificial Intelligence

Use of Artificial Intelligence: ChatGPT (OpenAI) was used for coding assistance. All code was reviewed, modified as needed, and executed locally by the authors.

## Conflict of interest

The authors declare there are no potential conflicts of interest concerning the research, authorship, and/or publication of this article.

## Declaration of interests

We declare no competing interests.

## References

[1] OECD Assessing the real cost of disasters: the need for better evidence. OECD; 2018. (OECD Reviews of Risk Management Policies). https://www.oecd.org/en/publications/assessing-the-real-cost-of-disasters_9789264298798-en.html.

[2] United Nations Office for Disaster Risk Reduction (2025). https://www.undrr.org/gar/gar2025.

[3] Bhola V., Hertelendy A., Hart A., Adnan S.B., Ciottone G. (2023). Escalating costs of billion-dollar disasters in the US: climate change necessitates disaster risk reduction. J Clim Change Health.

[4] Ebi K.L., Vanos J., Baldwin J.W. (2021). Extreme weather and climate change: population health and health system implications. Annu Rev Public Health.

[5] Gralewicz N.J., Nelson T.A., Wulder M.A. (2012). Factors influencing national scale wildfire susceptibility in Canada. Ecol Manag.

[6] Lévesque E. (2025). Exploring the geographical diversity of Canada: landscapes, climate, and human interaction. OTS Can J.

[7] Obani I.P., Obani Z.I., Anaeto F.C., Akroh T.O., Nwachukwu C.S. (2025). The economic costs of climate disasters: analyzing data from recent floods, wildfires, and hurricanes. Manuscript Sustain Econ Financ Manag.

[8] Subedi N. (2025). https://publications.gc.ca/collections/collection_2025/rncan-nrcan/Fo143-2-463-eng.pdf.

[9] Kunreuther H.C., Michel-Kerjan E.O., Doherty N.A., Grace M.F., Klein R.W., Pauly M.V. (2009). https://direct.mit.edu/books/book/3830/At-War-with-the-WeatherManaging-Large-Scale-Risks.

[10] Ghanbari-Jahromi M., Kharazmi E., Bastani P., Shams M., Marzaleh M.A., Amin Bahrami M. (2024). Factors disrupting the continuity of care for patients with chronic disease during the pandemics: a systematic review. Health Sci Rep.

[11] Feizolahzadeh S., Vaezi A., Mirzaei M. (2019). Barriers and facilitators to provide continuity of care to dischargeable patients in disasters: a qualitative study. Injury.

[12] Man R.X.G., Lack D.A., Wyatt C.E., Murray V. (2018). The effect of natural disasters on cancer care: a systematic review. Lancet Oncol.

[13] Hick J.L., Hanfling D., Cantrill S.V. (2012). Allocating scarce resources in disasters: emergency department principles. Ann Emerg Med.

[14] Daniell J.E. (2014). Earthquake Hazard, Risk and Disasters.

[15] Khan M.T.I., Anwar S., Sarkodie S.A., Yaseen M.R., Nadeem A.M. (2023). Do natural disasters affect economic growth? The role of human capital, foreign direct investment, and infrastructure dynamics. Heliyon.

[16] United Nations (2014). https://repositorio.cepal.org/server/api/core/bitstreams/ade10e58-089e-44e2-b781-5282e32687e7/content.

[17] Kousky C. (2014). Informing climate adaptation: a review of the economic costs of natural disasters. Energy Econ.

[18] Varner C. (2023). Emergency departments are in crisis now and for the foreseeable future. Can Med Assoc J.

[19] Varner C. (2023). Without more acute care beds, hospitals are on their own to grapple with emergency department crises. Can Med Assoc J.

[20] Uimonen M., Kuitunen I., Paloneva J., Launonen A.P., Ponkilainen V., Mattila V.M., Den Uil C. (2021). The impact of the COVID-19 pandemic on waiting times for elective surgery patients: a multicenter study. PLoS One.

[21] Martin D., Razak F., Bayoumi I. (2025). Primary care in the COVID-19 pandemic and beyond: lessons from Ontario. Can Fam Physician.

[22] Ford J.D., Berrang-Ford L., King M., Furgal C. (2010). Vulnerability of Aboriginal health systems in Canada to climate change. Glob Environ Change.

[23] Vodden K., Cunsolo A., Harper S.L., Kipp A., King N., Manners S. (2021). National Issues Report.

[24] Journeay M., Yip J.Z.K., Wagner C.L., LeSueur P., Hobbs T. (2022). https://ostrnrcan-dostrncan.canada.ca/handle/1845/134805.

[25] Shute J., Gamble A., Dieleman A. (2024). Indigenous housing and climate resilience report. Can Clim Inst.

[26] Hertelendy A.J., Burkle F.M., Ciottone G.R. (2022). Canadian wildfires: a plague on societies well-being, inequities and cohesion. Prehospital Disaster Med.

[27] Tymstra C., Stocks B.J., Cai X., Flannigan M.D. (2020). Wildfire management in Canada: review, challenges and opportunities. Prog Disaster Sci.

[28] Asfaw H.W., First Nation S.L., McGee T.K., Christianson A.C. (2019). A qualitative study exploring barriers and facilitators of effective service delivery for Indigenous wildfire hazard evacuees during their stay in host communities. Int J Disaster Risk Reduct.

[29] Coogan S.C.P., Robinne F.N., Jain P., Flannigan M.D. (2019). Scientists' warning on wildfire — a Canadian perspective. Can J For Res.

[30] MacLennan C. (2008). https://www.queensu.ca/iigr/sites/iirwww/files/uploaded_files/MacLenann.Chapter.pdf.

[31] Nikolakis W., Roberts E. (2022). Wildfire governance in a changing world: insights for policy learning and policy transfer. Risk Hazards Crisis Publ Pol.

[32] Clarke L., Patouillard E., Mirelman A.J., Ho Z.J.M., Edejer T.T.T., Kandel N. (2022). The costs of improving health emergency preparedness: a systematic review and analysis of multi-country studies. eClinicalMedicine.

[33] Agrawal N., Adhikari I., Yiu N. (2021). Disaster risk in Canada – a data-driven discussion. Can J Emerg Manag.

[34] Wang W., Wang X., Flannigan M.D. (2025). Canadian forests are more conducive to high-severity fires in recent decades. Science.

[35] Christianson A. (2015). Social science research on Indigenous wildfire management in the 21st century and future research needs. Int J Wildland Fire.

[36] Jain P., Barber Q.E., Taylor S. (2024). Canada under fire – drivers and impacts of the record-breaking 2023 wildfire season. https://essopenarchive.org/users/747500/articles/719254-canada-under-fire-drivers-and-impacts-of-the-record-breaking-2023-wildfire-season?commit=07df65f18343f98150852993157559888c17a1db.

[37] WHO (2016). An R&D blueprint for action to prevent epidemics. https://cdn.who.int/media/docs/default-source/blue-print/an-randd-blueprint-for-action-to-prevent-epidemics.pdf.

[38] CDC Public health emergency preparedness and response capabilities. https://www.cdc.gov/readiness/media/pdfs/CDC_PreparednesResponseCapabilities_October2018_Final_508.pdf.

[39] Sawyer D., Canadian Climate Institute Damage control: reducing the costs of climate impacts in Canada. https://climateinstitute.ca/wp-content/uploads/2022/09/Damage-Control_-EN_0927.pdf.

[40] Clark T.D., Willow Springs Strategic Solutions Natural disasters, vulnerability, and resilience in Indigenous communities: literature review and conceptual framework. Nat Disasters. https://www.researchgate.net/publication/318795788_Natural_Disasters_Vulnerability_and_Resilience_in_Indigenous_Communities_Literature_Review_and_Conceptual_Framework#fullTextFileContent.

[41] Intergovernmental Panel on Climate Change (Ipcc) (2023). https://www.cambridge.org/core/product/identifier/9781009157896/type/book.

[42] Hertelendy A.J., Maggin J., Ciottone G. (2025). Health care system adaptation and resilience during the wildfire crisis. JAMA.

[43] Hertelendy A.J., Dresser C., Gorgens S., Hertelendy A.P., Biddinger P.D., Ciottone G. (2025). Strengthening healthcare system resilience: a comprehensive framework for tropical cyclone preparedness and response. Lancet Reg Health Am.

[44] Hasan M.D.K., Nasrullah S.M., Quattrocchi A., Arcos González P., Castro-Delgado R. (2023). Hospital surge capacity preparedness in disasters and emergencies: a systematic review. Public Health.

[45] Sheikhbardsiri H., Raeisi A.R., Nekoei-moghadam M., Rezaei F. (2017). Surge capacity of hospitals in emergencies and disasters with a preparedness approach: a systematic review. Disaster Med Public Health Prep.

[46] Khushi S.R., Khoso A.R., Bhutto S., Narejo A.A. (2024). The long-term health impacts of repeated flood events: a review. J Environ Energy Econ.

[47] Howitt R., Havnen O., Veland S. (2012). Natural and unnatural disasters: responding with respect for Indigenous rights and knowledges. Geogr Res.

[48] Shannon G., Morgan R., Zeinali Z. (2022). Intersectional insights into racism and health: not just a question of identity. Lancet.

[49] Rose A., Prager F., Chen Z. (2017). http://link.springer.com/10.1007/978-981-10-2567-9.

[50] Watts N., Amann M., Arnell N. (2021). The 2020 report of the Lancet Countdown on health and climate change: responding to converging crises. Lancet.

