# Supplementary material for: Gaps in disaster cost reporting and rising economic burdens in Canada, 1990–2020: retrospective database analysis
Source: Lancet Reg Health Am. 2026 Mar 20;57:101445. doi: 10.1016/j.lana.2026.101445 (PMC13018982; doi:10.1016/j.lana.2026.101445)
Supplement: Supplementary Figures and Tables [file mmc1.docx]

**Gaps in Disaster Cost Reporting and Rising Economic Burdens in Canada, 1990–2020: Retrospective Database Analysis.**

Table of Contents

[Supplementary Results 2](#_Toc221632325)

[Supplementary table 1. 2](#_Toc221632326)

[Supplementary Table 2. 3](#_Toc221632327)

[Supplementary Figure 1. 4](#_Toc221632328)

[Supplementary Figure 2. 5](#_Toc221632329)

[Supplementary Figure 3 A–B. 6](#_Toc221632330)

# **Supplementary Results**

Numbers in front of each Table or Figure corresponds to the order it appears in the manuscript.

**1.** **Supplementary Table 1.** Annual frequency of meteorologic and wildfire disasters in Canada, 1990–2020.

| Hazard Type | Mean | SD | Median | IQR |
| --- | --- | --- | --- | --- |
| Meteorologic | 12.97 | 5.93 | 12 | 7.5 |
| Wildfire | 3.75 | 2.51 | 3 | 3 |

**2.** **Supplementary Table 2.** **Seasonal distribution of meteorologic and wildfire disasters, 1990–2020.**

| Season | Meteorologic | Wildfire |
| --- | --- | --- |
| Fall (September – November) | 63 | 4 |
| Winter (December – February) | 72 | 0 |
| Spring (March – May) | 114 | 29 |
| Summer (June – August | 153 | 57 |


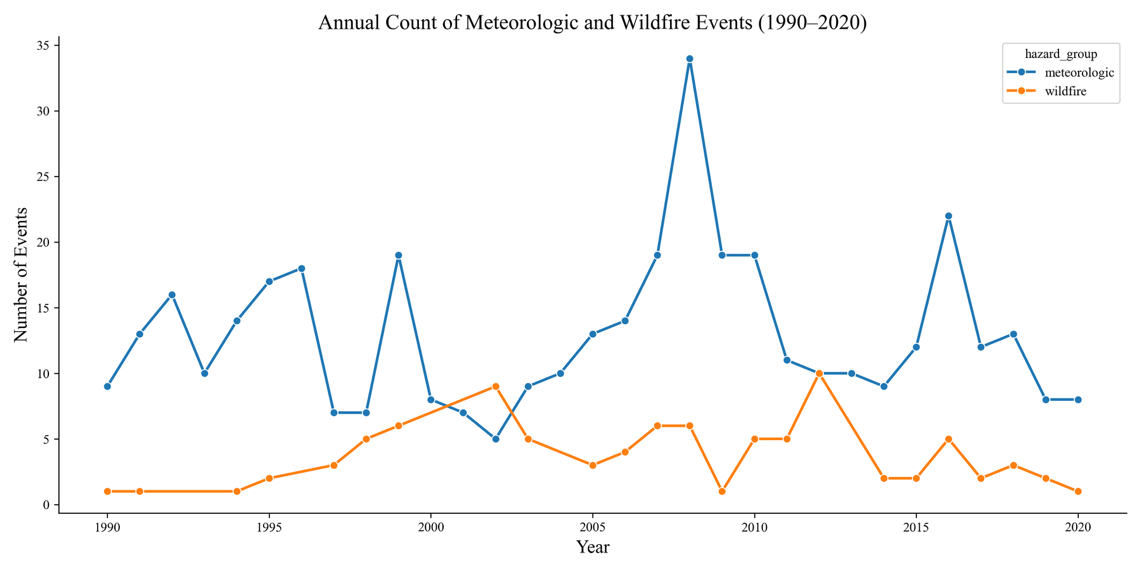


**3. Supplementary Figure 1.** **Annual counts of meteorologic and wildfire disasters recorded in Canada, 1990–2020.** This figure depicts the number of events per calendar year for each hazard type based on classifications in the Canadian Disaster Database, illustrating year-to-year variation in the frequency of meteorologic and wildfire disasters over the study period.


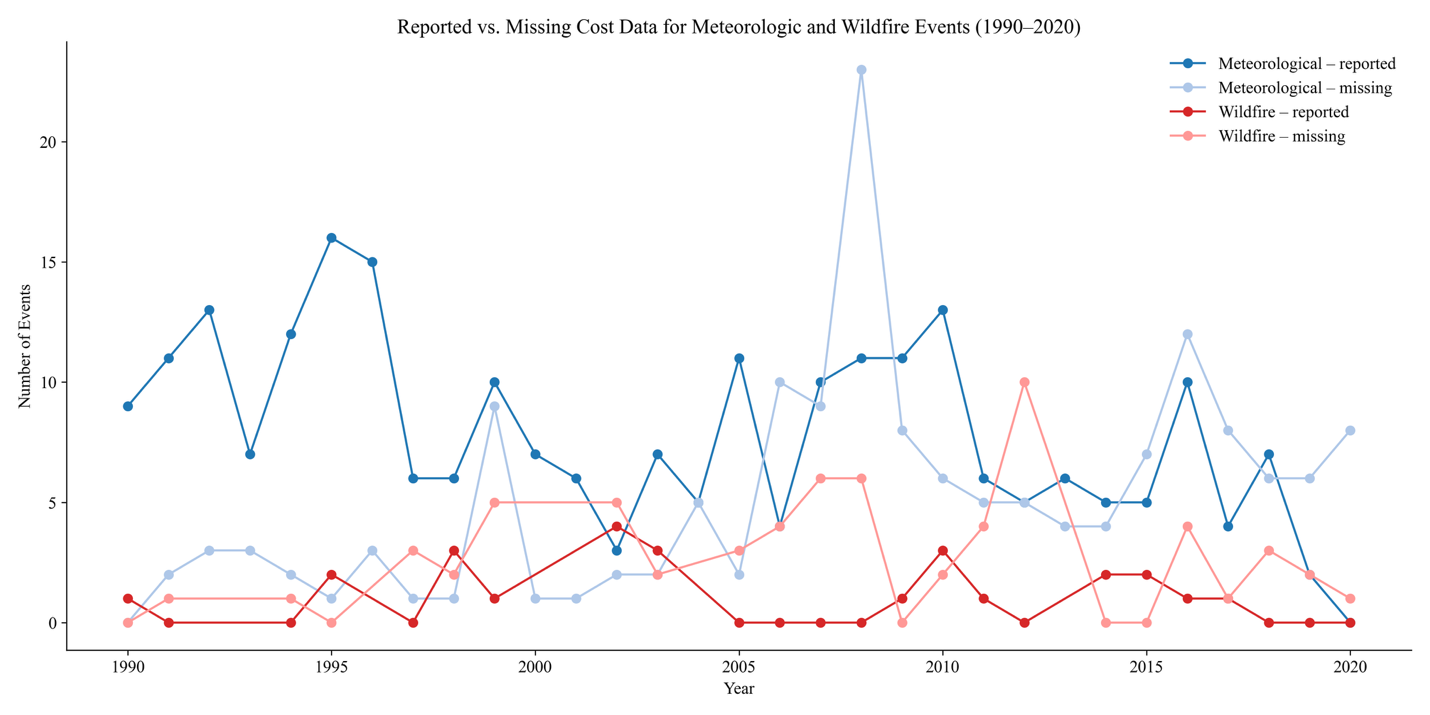


4. **Supplementary Figure 2.** **Reported vs. missing cost data for meteorologic and wildfire disasters in Canada (1990–2020).** Annual counts of meteorologic and wildfire events are shown stratified by whether economic cost estimates were reported in the Canadian Disaster Database. Meteorologic events remained frequent throughout the study period, but reporting completeness varied markedly, with a pronounced increase in missing cost data between approximately 2005 and 2010. This pattern corresponds to the apparent dip in meteorologic costs observed in the main analysis and indicates that the decline reflects gaps in cost reporting rather than a true reduction in disaster occurrence or disaster impacts.

**A.**
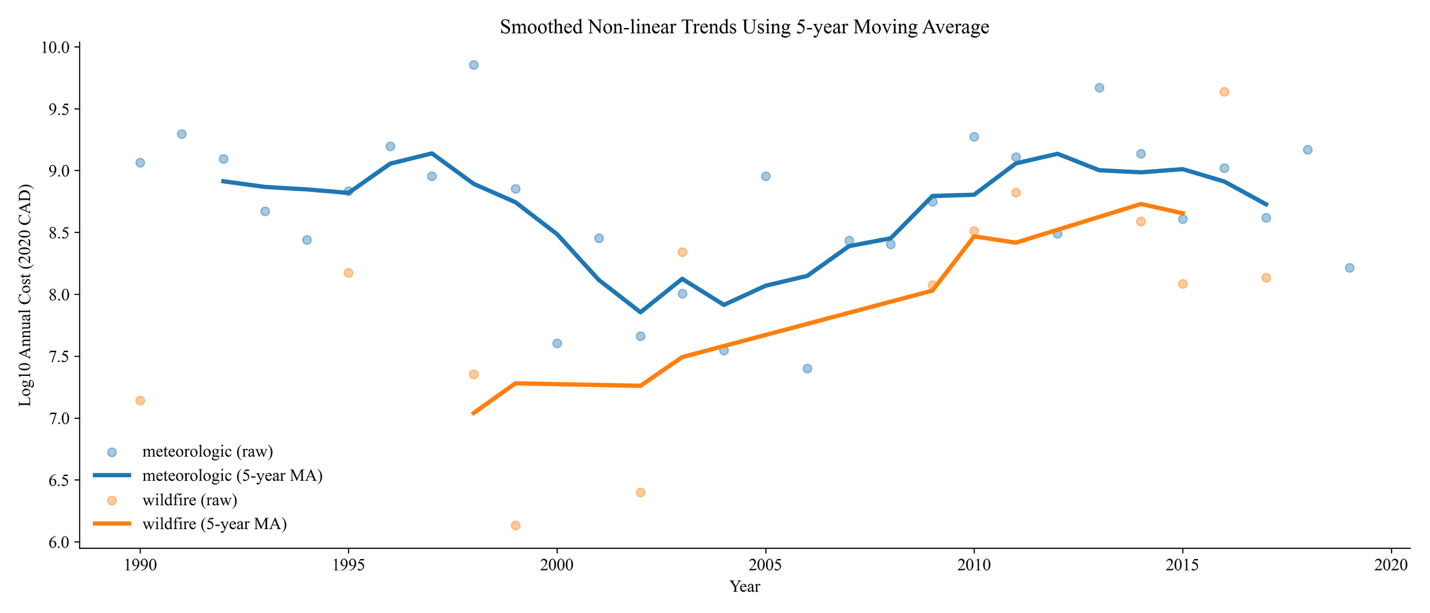


**B.**


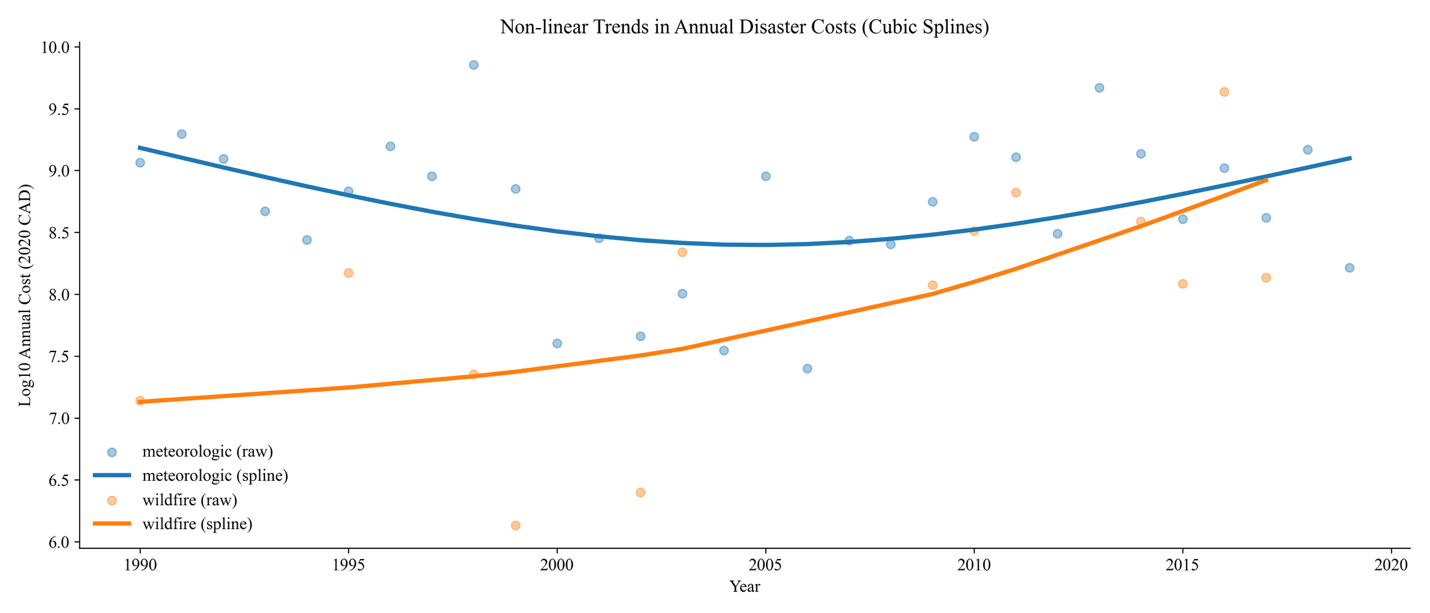


**5. Supplementary Figure 3.** A–B. **Non-linear visualizations of annual disaster-related costs for meteorologic and wildfire events in Canada, 1990–2020.** **(A)** Centered five-year moving averages of log-transformed annual costs, illustrating smoothed medium-term trends while reducing year-to-year variability. Wildfire costs show a sustained long-term increase, whereas meteorologic costs display modest curvature, an early 2000s decline followed by a gradual rise, without evidence of abrupt or persistent non-linear deviation. **(B)** Restricted cubic spline curves (3 degrees of freedom) showing directionally consistent with the moving-average trends and with the primary linear regression findings. This figure also shows progressive increases in wildfire costs and mild non-linear fluctuations in meteorologic costs without major departures from the overall upward trend.
